# Cardioembolic Stroke: A Matter of Prevention

**DOI:** 10.31083/j.rcm2401021

**Published:** 2023-01-11

**Authors:** Marialuisa Zedde, Rosario Pascarella

**Affiliations:** ^1^Neurology Unit, Department of Neuromotor Physiology and Rehabilitation, Azienda Unità Sanitaria Locale-IRCCS di Reggio Emilia, 42122 Reggio Emilia, Italy; ^2^Neuroradiology Unit, Department of Radiology, Azienda Unità Sanitaria Locale-IRCCS di Reggio Emilia, 42122 Reggio Emilia, Italy

Cardioembolic stroke represents a composite and heterogeneous etiopathogenetic category, including a wide range of diseases from atrial fibrillation (AF) to hypokinetic heart disease to takotsubo cardiomyopathy up to endocarditis and complicated aortic atheromatosis. Two reasons make this stroke subtype particularly relevant: first, stroke due to cardioembolism is more severe than stroke due to other etiologies [1]; second, the incidence of cardioembolic stroke is rising despite an overall decrease of stroke incidence in high-income countries [2]. Cardioembolism globally accounts for about 25% of ischemic strokes [3] and a main role is played by AF, if only for its prevalence in an aging population and for its therapeutic implications in primary and secondary prevention. Indeed, AF causes at least an half of cardioembolic strokes and, in a population setting, one of the strongest risk factors for the development of AF is age for both genders, as evident in the Rottherdam Study [4]. The overall prevalence of AF was 5.5%, rising from 0.7% in the age group 55–59 years to 17.8% in those aged 85 years and above and the lifetime risk to develop AF at the age of 55 years was 23.8% in men and 22.2% in women [4]. This issue has an impact on the evaluation of benefit-to-risk ratio for anticoagulant treatment, which is a paramount stone of the prevention in AF patients. Unfortunately, both the bleeding and the embolic risk increase proportionately with the increasing age both in patients taking vitamin K antanonists (VKAs) and in patients taking directs anticoagulants (DOACs), in real life and in clinical trials. Moreover, the bleeding risk of patients taking DOACs cannot be reliably estimated using the HAS-BLED score and, obviously, it includes not only brain bleeding, which is accociated with a severe prognosis whatever anticoagulant was taken, but also systemic bleeding events. Apart from the well known increase of gastrointentinal bleeding in patients taking DOACs, real life data comparing the safety profile of the individual DOACs are substantially missing. The systematic review of Archontakis-Barakakis *et al*. [5] addresses this issue considering major hemorrhages (MH) in patients anticogulated with VKAs and DOACs for non valvular AF. The authors found that the MH risk associated with Rivaroxaban use was higher than the risk with Dabigatran use [HR (hazard ratio): 1.32, 95% CI (confidence interval): 1.21–1.45] but similar to VKA use (HR: 0.94, 95% CI: 0.87–1.02) and the MH risk associated with Apixaban use was lower than the risk with Dabigatran use (HR: 0.75, 95% CI: 0.64–0.88) [5]. These data might be particularly relevant in comorbid and elderly patients, although some issues in increasing the risk of intracranial bleeding (e.g., small vessel disease neuroimaging markers) are not included.

When an acute ischemic stroke occurs, in particular when the patient is taking an anticoagulant drug, one of the main reperfusive treatment is the endovascular approach, well summarized in the review of Bucke *et al*. [6]. The endovascular treatment of acute stroke gained a strong evidence of efficacy and the indications are progressively expanding, both because of an extension of the time window from symptoms onset and the treatment of distal vessels through a tumultuous development of technologies and materials. Moreover, novel techniques are developing, such as the direct aspiration first-pass technique (ADAPT) or a combined stent retriever and distal aspiration approach. In this regard, if AF has no effect in the successful rate of endovascular treatment and no difference in outcome between large vessel occlusion stroke patients with and without AF was demonstrated in a large meta-analysis [7], thrombus histhology may help to define the etiology of ischemic stroke and in particular to orient to a cardioembolic source [8]. These data are perfectly coherent with the finding that the histhology of thrombi from patients with stroke of suspected cardioembolic origin and prior anticoagulant therapy does not differ from those without prior anticoagulant therapy, but both differ from non-cardioembolic thrombi [9].

Despite its efficacy in preventing ischemic stroke and systemic embolism in AF, anticoagulant drugs are still underused and the development of strategies to increase the awareness anf the compliance of patients in taking the therapy is one of the main issue, analyzed in the systematic review of Baers *et al*. [10]. In particular, patient decision aids may be effective on the choice of and adherence to stroke prevention therapy in individuals with AF, but they have a variable impact on it and have to be integrated and implemented.

Meanwhile, several well designed population studies aimed to find strong and easily achievable biomarkers to predict the prognosis of stroke patients in order to better plan the pathways of care. In the National Health and Nutrition Examination Survey (NHANES-III) study [11] one of these biomarkers was serum 5 beta-2 microglobulin (B2M), tested in 4914 US adults (mean age = 63.0 years, 44.3% male) followed-up for a median of 19.4 years. The authors found that B2M may be a marker of stroke and all-cause mortality but the study did not provided a separate analysis according to the etiology of stroke.

Several fields are still incompletely explored and therefore the evidence level is low. For example the treatment of embolic sources different from AF has not been defined in the same way as AF and conditions as low systolic function, aortic arch atheromas, valvular heart diseases, dilated cardiopathy have different implications for the choice of antithrombotic therapy. Moreover, the incorporation of neuroimaging items in composite scores for predicting embolic and hemorrhagic risk in stroke patients with different antithrombotics is a promising strategy [12], but further evidence is needed.
